# The Physician Himself, and What He Should Add to His Scientific Acquirements in Order to Secure Success

**Published:** 1886-06

**Authors:** 


**The Physician Himself, and what he should add to his Scientific Acquirements in order to secure Success.** By D. W. CATHELL, M. D., late Professor of Pathology in the College of Physicians and Surgeons of Baltimore. Sixth edition, thoroughly revised. Baltimore: Cushings & Bailey, 262 West Baltimore street. 1885.

"The numerous commendatory notices of the work by the medical press, the encomiums of physicians in every section of our country, and the rapid sale of five large editions, are gratifying proof that a want of such an essay was felt by many members of the profession. Grateful for this result, and desiring to render the volume worthy of the favor with which it has been received, the author, in preparing the sixth edition, has made such alterations and additions as greater experience and more mature reflection have dictated." We can only say what we have already said of former editions of this work, that it is indispensable to every physician, as it gives numberless hints concerning "professional tact and business sagacity" which the most of them lack.

